# A Comprehensive Guide to Implement Artificial Intelligence Cloud Solutions in a Dental Clinic: A Review

**DOI:** 10.7759/cureus.78718

**Published:** 2025-02-07

**Authors:** Sumit V Bedia, Murtaza Akbarali Shapurwala, Bhushan Pramod Kharge, Aarti S Bedia, Amit Patil

**Affiliations:** 1 Prosthodontics, Bharati Vidyapeeth (Deemed to be University) Dental College and Hospital, Navi Mumbai, IND; 2 Dentistry, Bharati Vidyapeeth (Deemed to be University) Dental College and Hospital, Navi Mumbai, IND; 3 Oral Medicine and Radiology, Bharati Vidyapeeth (Deemed to be University) Dental College and Hospital, Navi Mumbai, IND; 4 Conservative Dentistry and Endodontics, Bharati Vidyapeeth (Deemed to be University) Dental College and Hospital, Navi Mumbai, IND

**Keywords:** artificial intelligence in dentistry, cloud computing, data security, patient data management, staff education

## Abstract

Integrating the artificial intelligence (AI) cloud into dental clinics can enhance diagnostics, streamline operations, and improve patient care. This article explores the adoption of AI-powered cloud solutions in dental clinics, focusing on infrastructure requirements, software licensing, staff training, system optimization, and the challenges faced during implementation. It provides a detailed guide for dental practices to transition to AI cloud systems. We reviewed existing literature, technological guidelines, and practical implementation strategies for integrating AI cloud in dental practices. The methodology includes a step-by-step approach to understanding clinic needs, selecting appropriate software, training staff, and ensuring system optimization and maintenance. Integrating AI cloud solutions can drastically improve clinical outcomes and operational efficiency. Despite the challenges, proper planning, infrastructure investment, and continuous training can ensure a smooth transition and maximize the benefits of AI technologies in dental care.

## Introduction and background

Cloud computing is the delivery of servers, storage, databases, networking, software, and analytics over the Internet to enable faster innovation, flexible resources, and economies of scale [1]. Artificial intelligence (AI) cloud combines the power of AI with the scalability and flexibility of cloud computing. The rapid development of AI technology is built on three key cornerstones: big data, computational power, and advanced AI algorithms. Big data, generated through digital devices and online interactions, provides the vast information needed to train AI systems. Increased computational power, driven by innovations in cloud computing, enables the processing of these large datasets efficiently. Meanwhile, advancements in AI algorithms, such as deep learning, have enhanced the ability of systems to learn, adapt, and solve complex problems. By leveraging platforms like Amazon Web Services (AWS), Google Cloud, and Microsoft Azure, AI cloud allows dental professionals to process large datasets, including patient records, imaging data, and diagnostic results, without extensive on-site hardware. This technology enables the deployment of AI-powered tools such as diagnostic aids, patient management systems, and treatment planning applications. With capabilities like real-time analytics, image recognition, and predictive modeling, AI cloud is revolutionizing the way dental clinics operate and deliver care [2-3].

In dental clinics, AI cloud is integrated into various aspects of practice, from enhancing diagnostics to streamlining workflows. It powers advanced imaging systems that analyze radiographs, cone-beam computed tomography (CBCT) scans, and panoramic images for the early detection of conditions like caries and periodontal disease [4]. In addition, AI tools assist in creating 3D models for prosthetics and orthodontics, while also providing real-time diagnostic feedback for faster clinical decision-making [5]. Cloud-based patient management systems optimize scheduling, automate billing and insurance claims, and centralize patient data for easy access [6]. Moreover, AI enhances patient engagement through virtual consultations, personalized education tools, and predictive treatment planning. By automating routine tasks and supporting research, AI cloud is not just improving patient care but also making dental clinics more efficient and future-ready [7].

## Review

Step 1: understanding requirements

For a dental clinic incorporating cloud-based systems, a reliable and fast Internet connection is crucial, with a minimum speed of 10 Mbps for basic tasks like scheduling and billing. However, for cloud-based imaging, X-ray storage, 3D scans, and real-time CAD/CAM data transfer, 25-50 Mbps per device are needed and up to 100 Mbps for seamless clinic-wide operations. Fiber-optic connections with backup options ensure consistent performance [8]. Essential hardware includes five to seven devices, such as mobile/tablet PCs for reception, devices at each dental chair for imaging and treatment planning, and backup devices for peak times or technical issues [9]. For cloud services, platforms like Dentrix Ascend and Curve Hero offer scalable subscription models that include data storage, software updates, and technical support, with maintenance costs lower than traditional IT setups [10]. Data security is vital, with compliance with the Accountability Act (HIPAA) and General Data Protection Regulation (GDPR) through encryption, role-based access, audit trails, regular backups, and secure storage. Clinics should also adopt strong cybersecurity practices, employee training, and endpoint security to safeguard patient data and ensure continuous compliance [11].

Step 2: software licensing

The licensing procedure for installing AI-powered software in a dental clinic involves selecting suitable software, choosing between subscription-based or one-time licensing models, and signing a contractual agreement that outlines usage terms. Once licensed, the software is installed, either via cloud-based or on-premises methods, and integrated with existing systems like electronic health records (EHR) [12]. Clinics usually receive training on software usage as part of the license, while ongoing support and updates are provided by the vendor to ensure compliance and functionality. These steps ensure the software meets regulatory standards and integrates smoothly into clinic operations [3].

Step 3: data migration

Data migration is a critical phase when incorporating AI-powered cloud solutions into a dental clinic. It involves transferring existing patient records, medical histories, diagnostic images, and other vital data from legacy systems (e.g., paper-based records or outdated EHR systems) into the new AI cloud infrastructure. Successful data migration requires careful planning, robust backup strategies, and ensuring compliance with data security regulations like HIPAA and GDPR. Breaching these regulations can cause significant operational disruptions, redirecting resources to manage and investigate the breach. Compromised data integrity may lead to incorrect diagnoses, inappropriate treatments, and delays in patient care, directly affecting patient safety. In addition, a breach can erode patient trust, making individuals hesitant to share their health information in the future, which could hinder effective care delivery. Legal consequences are severe, with patients having the right to file lawsuits for mishandling of their data under both HIPAA and GDPR. GDPR also allows individuals to seek compensation through legal action. Healthcare organizations must prioritize data security to avoid these legal and financial repercussions. A breach may require mandatory notifications to affected individuals and regulators, leading to public awareness and reputational damage. This public scrutiny increases the long-term oversight of healthcare organizations, making regulatory compliance even more critical. Therefore, safeguarding sensitive health data is not only necessary to avoid penalties but also to maintain trust and integrity in healthcare systems. Data integrity must be maintained during this transition, as errors in data migration can lead to significant clinical and legal consequences [13]. The first step in data migration is assessing the current data formats, volumes, and the compatibility of existing records with the new AI cloud system. This ensures that data is properly formatted, cleaned, and organized for optimal integration. To minimize disruptions during migration, clinics should conduct this process during off-peak hours and test the new system's functionality with a sample of migrated data before fully transitioning. Once the migration is complete, regular audits should be conducted to verify the accuracy of the data and the functionality of the AI tools within the cloud system. As per HIPAA, regular risk assessments and audits are recommended at least annually or whenever there are significant system changes. Similarly, GDPR emphasizes conducting regular reviews and assessments to ensure compliance with data protection principles, though it does not mandate a specific schedule, leaving the frequency to the organization's discretion. Continuous data backup is essential to safeguard patient information throughout this process [14].

Step 4: staff training

For the successful adoption of AI-powered software in a dental clinic, a multidisciplinary team is essential. Dentists and clinicians need training to interpret AI-driven diagnostic insights and integrate them into clinical decision-making, ensuring AI complements their expertise [15]. Dental technicians should understand how AI integrates with imaging and CAD/CAM systems, focusing on the accuracy and limitations of AI predictions in diagnostics and treatment planning [16]. Administrative staff must become proficient in managing patient data, appointments, and treatment plans, optimizing the clinic’s efficiency with AI-driven automation [17]. Data security personnel require specialized knowledge in data encryption, access control, and audit trails to ensure compliance with HIPAA or GDPR standards, safeguarding patient confidentiality [11]. In addition, IT support teams must be equipped to troubleshoot issues, manage software updates, and integrate AI systems with existing clinic technologies to avoid workflow disruptions [18]. Ongoing training is crucial to keep the team updated on software developments and new AI features, ensuring the clinic maximizes AI technology's potential in improving clinical outcomes and operational efficiencies [19].

Step 5: software optimization and maintenance

It is crucial to ensure regular updates for both new features and security enhancements. Automatic updates should be enabled to maintain security compliance and take advantage of emerging functionalities [20]. Introducing new features involves pilot testing with staff, followed by targeted training to ensure seamless integration into daily operations [21] Quarterly performance assessments, focusing on system efficiency, user satisfaction, and feedback, should be conducted to identify areas for improvement and optimize software utilization [22].

Challenges

Adopting AI-based cloud systems in dental clinics presents several challenges that must be addressed for successful integration. Infrastructure limitations are a major barrier, especially in smaller cities and rural areas where clinics may lack consistent internet connectivity and the hardware required to support AI systems [23]. These issues can result in inefficiencies and disruptions when operating cloud-based systems. Furthermore, data security and privacy concerns remain a significant challenge, as dental clinics must comply with national data protection regulations [24]. Many clinics still rely on paper-based records or outdated digital systems, making it difficult to ensure data protection, especially during the transition to digital or AI platforms [25]. Cost is another significant challenge, as the initial investment in AI technologies, including software licensing, cloud subscriptions, hardware, and training, can be prohibitively high for small and mid-sized clinics. In addition, the ongoing operational costs for software updates and maintenance add a financial burden [26]. This is compounded by the lack of technological literacy among staff, leading to resistance to change and fear of job displacement. Integration with existing systems is also a challenge, as AI-based systems may need to be compatible with older clinic technologies like EHRs, diagnostic tools, or imaging software. This integration process can be time-consuming and may require additional customization, increasing both costs and complexity. Moreover, the accuracy and reliability of AI algorithms in clinical settings are a concern, as these systems must provide accurate and dependable outputs for diverse dental cases to gain practitioners' trust. Clinicians are often reluctant to rely solely on AI for diagnosis and treatment recommendations [27]. Lastly, regulatory challenges exist due to the nascent nature of AI in healthcare. The lack of clear guidelines on the legal liability associated with AI errors or misdiagnosis can create hesitation in adopting AI systems. Clinics may worry about the legal implications if AI-generated recommendations lead to adverse outcomes [28].

Key performance indicators (KPIs)

Table 1 provides an overview of key platforms categorized by their applications and core features, which provide a roadmap for clinics looking to enhance their operational and clinical capabilities using AI and cloud-based systems [29-40]. KPIs are crucial for evaluating the success of AI-powered cloud systems in dental clinics. System uptime ensures uninterrupted operations, enabling real-time access to patient records and efficient scheduling. Data security compliance with frameworks like HIPAA and GDPR safeguards sensitive patient information through encryption and secure access controls. Financial efficiency is achieved via subscription-based models, reducing costs linked to hardware, licensing, and maintenance [11]. Patient satisfaction improves with shorter wait times, faster record access, and streamlined communication. AI’s diagnostic accuracy and treatment planning capabilities enhance clinical outcomes, while automation boosts staff productivity by reducing time spent on non-clinical tasks. Effective training ensures seamless user adoption, minimizing resistance to change. Lastly, scalable and flexible cloud systems support clinic growth without heavy infrastructure costs. By tracking these KPIs, clinics can achieve operational efficiency, superior care delivery, and long-term innovation [22]. 

**Table 1:** Smart dental clinics: applications and platform features

Application	AI platform	Key features	Reference
Diagnostics and imaging	Overjet	1. X-ray analysis	[29]
		2. Patient-friendly visuals	
		3. Practice integration	
		4. FDA-approved	
	Pearl	1. AI diagnostics	[30]
		2. Cloud storage	
		3. Automated workflows	
		4. User-friendly	
Patient management	CareStack	1. All-in-one dental software	[31]
		2. Automated scheduling	
		3. Integrated billing and charting	
	Open Dental Cloud	1. Centralized patient records	[32]
		2. Third-party integration	
		3. Affordable for clinics	
	YAPI	1. Automated communication	[33]
		2. Paperless patient forms	
		3. Software integration	
Billing and insurance	DentalXChange	1. Insurance claims management	[34]
		2. Real-time updates	
		3. Patient billing solutions	
	Simplifeye	1. Payment collection tools	[35]
		2. Patient financing	
		3. Streamlined billing	
Cloud data storage	Google Cloud AI	1. Custom ML models	[36]
		2. HIPAA-compliant atorage	
		3. Scalable for any practice	
	Microsoft Azure AI	1. AI-powered solutions	[37]
		2. Telehealth support	
		3. Advanced customization	
	AWS HealthLake	1. Centralized data storage	[38]
		2. Imaging workflows	
		3. Cost-effective and scalable	
Telehealth	Teledentix	1. Virtual consultations	[39]
		2. Secure data storage	
		3. Practice integration	
	MouthWatch TeleDent	1. Secure video consultations	[40]
		2. Portable intraoral cameras	
		3. HIPAA-compliant data	

Critical consideration for AI platforms in dental practice management

AI-powered platforms have transformed dental practices by introducing efficiency, accuracy, and enhanced patient engagement. Overjet, for example, leverages AI for X-ray analysis, delivering faster and more precise diagnostics. Its ability to integrate seamlessly with existing practice management systems and its FDA approval make it a reliable choice [29]. However, the high upfront cost, dependency on specific imaging equipment, and the occasional need for advanced hardware pose challenges for smaller or resource-constrained clinics. Furthermore, practitioners may require training to interpret AI-generated visuals effectively and incorporate them into patient communication, especially when explaining complex findings [26]. Similarly, Pearl enhances diagnostic accuracy through AI-driven support and offers cloud storage for easy data accessibility and automated workflows. While its user-friendly design boosts efficiency [30], the platform faces challenges related to a steep learning curve for integrating AI diagnostics. The possibility of false positives or misinterpretations of AI-driven analyses could lead to clinician hesitation, requiring ongoing validation and trust-building among users [28].

In terms of patient management, CareStack provides an all-in-one solution integrating scheduling, billing, and charting, reducing administrative burdens [31]. However, the platform's complexity and high cost can be overwhelming for smaller practices or those transitioning from simpler systems [23]. On the other hand, Open Dental Cloud, while more affordable and offering centralized patient records [32], has limited advanced features and an outdated user interface that some practitioners find cumbersome. YAPI excels in automating communication and offering paperless patient forms, making administrative tasks easier [33], but its lack of customizability and time-consuming setup process can hinder adoption in practices requiring tailored solutions [27]. For billing and insurance, platforms like DentalXChange streamline claims management and integrate billing processes efficiently [34]. However, clinics often face challenges such as extensive training requirements to maximize the platform’s utility and additional fees, which can add to operational costs [26]. Similarly, Simplifeye simplifies payment collection and offers financing options, but its focus on billing alone necessitates additional tools for broader practice management [35]. Moreover, transaction fees can accumulate, adding to financial strain for smaller practices.

When it comes to cloud data storage, platforms like Google Cloud AI and Microsoft Azure AI provide HIPAA-compliant solutions with customizable machine learning models [36,37]. While these platforms offer scalability and robust AI capabilities, their high setup costs and the requirement for skilled IT professionals pose significant barriers for clinics without dedicated technical staff. In addition, AWS HealthLake, while cost-effective and scalable, is often considered complex and intimidating for smaller practices, especially those unfamiliar with centralized medical data storage workflows [38]. In the realm of telehealth, platforms like Teledentix enable virtual consultations with secure data storage and easy practice integration [39]. However, its limited features and dependence on a reliable Internet connection can disrupt service delivery in clinics with inconsistent connectivity [8]. These platforms enhance remote consultations with portable intraoral cameras and secure video tools, yet the costs associated with the hardware and the limited integration with broader practice management systems present challenges, particularly for clinics seeking all-encompassing solutions.

## Conclusions

Incorporating AI cloud into dental clinics not only enhances operational efficiency but also significantly elevates patient care. By automating routine tasks, AI allows dental professionals to dedicate more time to direct patient interactions, improving overall service delivery. The accuracy and speed of AI-powered diagnostic tools ensure that dental conditions are detected early, leading to more effective treatments and better patient outcomes. The personalized, faster service enabled by AI leads to a more satisfying patient experience, with improved communication and tailored care plans. Embracing AI cloud not only transforms the operational aspects of a dental clinic but also sets the foundation for future innovation, ensuring that both clinics and patients benefit from the cutting-edge advancements in dental healthcare.
